# Multi-Focal Laser Direct Writing through Spatial Light Modulation Guided by Scalable Vector Graphics

**DOI:** 10.3390/mi14040824

**Published:** 2023-04-07

**Authors:** Linhan Duan, Yueqiang Zhu, Haoxin Bai, Chen Zhang, Kaige Wang, Jintao Bai, Wei Zhao

**Affiliations:** State Key Laboratory of Photon-Technology in Western China Energy, International Collaborative Center on Photoelectric Technology and Nano Functional Materials, Institute of Photonics & Photon Technology, Northwest University, Xi’an 710127, China

**Keywords:** two-photon lithography, scalable vector graphics, spatial light modulator, multi-focus parallel processing

## Abstract

Multi-focal laser direct writing (LDW) based on phase-only spatial light modulation (SLM) can realize flexible and parallel nanofabrication with high-throughput potential. In this investigation, a novel approach of combining two-photon absorption, SLM, and vector path-guided by scalable vector graphics (SVGs), termed SVG-guided SLM LDW, was developed and preliminarily tested for fast, flexible, and parallel nanofabrication. Three laser focuses were independently controlled with different paths, which were optimized according to the SVG to improve fabrication and promote time efficiency. The minimum structure width could be as low as 81 nm. Accompanied by a translation stage, a carp structure of 18.10 μm × 24.56 μm was fabricated. This method shows the possibility of developing LDW techniques toward fully electrical systems, and provides a potential way to efficiently engrave complex structures on nanoscales.

## 1. Introduction

Two-photon lithography [1,2,3,4] is one of the most widely used techniques in the field of micro-/nanofabrication, e.g., in semiconductors [5], micro-nanofluidics [6,7,8], metamaterials [9], optical information storage [10], and biomedical engineering [11,12,13]. The photosensitive molecules in the material absorb photons through two-photon absorption and initiate subsequent photopolymerization chemical reactions, which is referred to as two-photon polymerization (TPP). This is a unique micro-fabrication technique that utilizes the nonlinear relationship between the polymerization rate and radiant light intensity to produce true three-dimensional structures with feature sizes below the diffraction limit [14,15]. Single-point TPP lithography was initially advanced to realize the fabrication of 3D structures [16]. The method is relatively simple and can be achieved by tuning either the galvanometer or translation stage [17]. However, single-point TPP lithography techniques are normally time-consuming with low fabrication efficiency.

To overcome these shortcomings, a series of methods using multi-focal spots to realize parallel fabrication have been developed in the last decade. Multi-focal spots can be generated in a variety of ways, including using fixed and variable elements. Fixed elements can only produce multi-focal spots with fixed numbers, positions, and sizes [18,19]. The representative approaches are through a micro-lens array [20,21], laser interferometry [22,23], etc. However, these methods can only achieve high-throughput fabrication of the identical structures by moving the galvanometer and translation stage.

Compared to fixed elements, variable elements can produce flexible and controllable multi-focal spots [24,25,26]. Among them, the beam-shaping method based on spatial light modulation (SLM), which can modulate the phase of incident light, can generated multiple, diverse, and flexibly distributed beams. This provides a high-efficiency and flexible approach for laser fabrication. There are various methods for generating multi-focal spots based on SLM [27,28,29], such as the Gerchberg–Saxton (GS) algorithm [30,31], the weighted Gerchberg–Saxton (GSW) algorithm [32], the adaptive additive (AA) algorithm [33], and the strip segmentation phase (SSP) [34] method. The GS, GSW, and AA algorithms are iterative algorithms, which normally take a long time in the generation of phase maps. Meanwhile, the modulated beams generated by applying phase maps through these iterative algorithms are normally nonuniform with undesired ripples. In contrast, the SSP method, which is a noniterative method, can generate high-quality phase maps for modulating multi-focal spots with uniform and diverse beams. This method provides an effective approach to realize the fast modulation of beams for SLM-based two-photon laser direct writing.

In laser direct writing (LDW) techniques, the arrangement of the path is important in improving the efficiency and quality of fabrications. There are some commonly used path arrangement methods in LDW, such as progressive scanning [35], zigzag scanning [36], and layer-by-layer scanning [37] for 3D structures. For instance, Vizsnyiczai et al. [38] developed a multi-focus fabrication method realized by SLM. The phase maps were designed by the GSW algorithm, and the paths of the laser focuses were arranged based on the coordinate of the target. First, a multi-dodecahedron was virtually built up using OpenGL software (4.4, Khronos Group, Santa Clara, CA, USA) and converted to voxel coordinates. Then, the voxels were manually assigned to each laser focus “ergodically”. Wu et al. [39] proposed a parallel fabrication method with a multi-focus array obtained via superposing opposite-ordered Bessel beams. The focal pattern and focal spot position could be rearranged by changing the order of the holograms. However, these methods have the same issues, in that the path should be arranged and optimized manually.

The simultaneous high flexibility and time efficiency of TPP lithography with high resolution is what all scholars have been pursuing. In this investigation, we propose a novel two-photon laser direct writing method named SVG-guided SLM LDW. By combining SLM to control multi-focus movements and vector graphics to arrange the path, we hope to realize an independent multi-focus and flexible, high-throughput nanoscopic fabrication. We hope that this preliminary investigation can provide a new direction for the development of SLM-based two-photon laser direct writing.

## 2. Method

**Figure 1:**
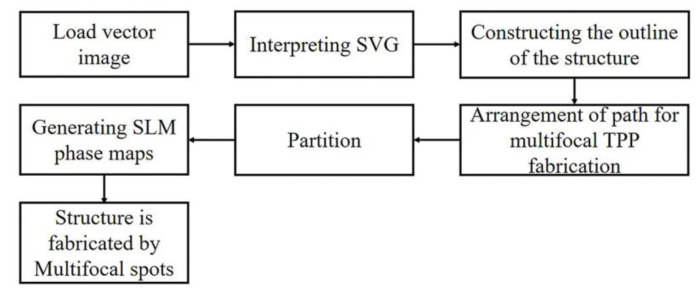
Schematic diagram of the fabrication of large complex vector structures.

The method is schematically diagrammed in Figure 1. In this method, the fabrication of large complex structures is carried out in parallel by multi-focal spots, generated, and moved through SLM. The path of each laser spot is arranged according to the vector graphic of the fabricated structure.

### 2.1. Multi-Focus Generated by SLM

**Figure 2:**
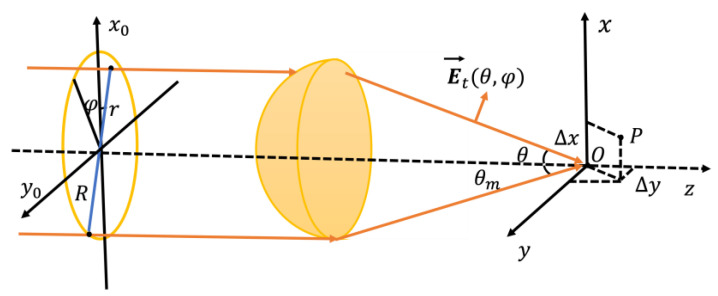
Schematic of the focused beam after passing through the objective. Here, φ is the azimuthal angle of the objective, r is polar coordinate, θ is convergence angle of the objective, R is the maximum radius of the pupil plane, and $\theta_{m}$ is the maximum convergence angle of the objective.

As is shown in Figure 2, the electric field distribution at an arbitrary point in the focal region of a high numerical aperture (NA) aberration-free lens can be calculated by Debye diffraction theory [40,41,42,43], which is as follows:

(1) $$E\left(x,y,z\right)=\int_{0}^{\theta }\int_{0}^{2\pi }P\left(\theta \right)E_{t}\left(\theta ,\varphi \right)\times \exp\left\{-ik\sqrt{x^{2}+y^{2}}\sin\theta \cos\left[\tan^{-1}\left(y/x\right)-\varphi \right]\right\}\;\times \exp\left(ikz\cos\theta \right)\sin\theta d\theta d\varphi \;=\iint \frac{P\left(\theta \right)E_{t}\left(\theta ,\varphi \right)}{\cos\theta }e^{ik_{z}z}e^{i2\pi \left(\xi x+\eta y\right)}d\xi d\eta \;\;=F^{-1}\left[P\left(\theta \right)E_{t}\left(\theta ,\varphi \right)e^{ik_{z}z}/\cos\theta \;\right]$$

where $P\left(\theta \right)$ is the pupil function of the objective, $E_{t}\left(\theta ,\varphi \right)$ is the transmission electric field, φ is the azimuthal angle of the objective, $\theta =\arcsin\left(\frac{rNA}{Rn_{t}}\right)$ is the convergence angle of the objective, NA is the numerical aperture of the objective, R is the maximum radius of the pupil plane behind the objective, $\theta_{m}$ is the maximum θ, $n_{t}$ is the refractive index of the objective, ξ=cosφsinθ/λ and η=sinφsinθ/λ denote the spatial frequency in x and y directions, λ is the wavelength in vacuum, and $k_{z}$ is the wave vector in the z direction. Here, we assume that the optical system follows the Abbe sinusoidal condition. The coordinate r is the polar coordinate in the pupil aperture plane, while x, y, and z are the Cartesian coordinates of the focal region.

According to Equation (1), the electric field after modulation is as follows:

(2) $$E\left(x,y,z\right)=F^{-1}\left[e^{i\psi }P\left(\theta \right)E_{t}\left(\theta ,\varphi \right)e^{ik_{z}z}/\cos\theta \;\right]$$

where ψ is the phase distribution function, which can be expressed as follows:

(3) $$\psi \left(x_{0},y_{0}\right)=\frac{2\pi }{\lambda }\frac{NA}{Rn_{t}}\left(x_{0}\Delta x+y_{0}\Delta y\right)$$

where λ is the laser wavelength, $x_{0}$ and $y_{0}$ are the coordinates of the pupil aperture plane of the objective, and Δx and Δy are the relative displacement components relative to the original focus of the objective in the x and y directions of the focal plane.

### 2.2. Extracting the Path from the SVG

**Figure 3:**
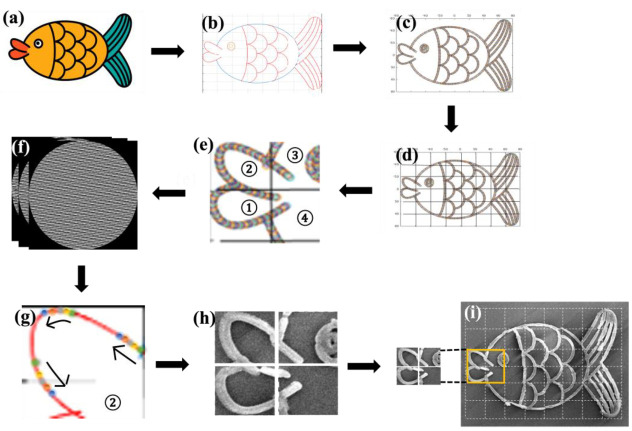
SVG decoding and fabrication flow chart with a carp structure as an example. (**a**) Interpret the SVG into mathematical equations, with a carp pattern as example. (**b**) Construct the outline of the carp. (**c**) Discrete the outline into coordinate points ($X\left(l\right)$, *Y*$\left(l\right)$). (**d**) Divide the carp structure into different partitions depending on the target size. (**e**) Rearrange the coordinate points ($X_{i}\left(l\right)$, $Y_{i}\left(l\right)$) in the partitions, e.g., ①, ②, ③, ④. (**f**) Generate phase maps with three Bessel–Gaussian laser spots and their movements. (**g**) According to the coordinates through path designing, where the arrows represent the direction of the fabrication path. (**h**) Local fabrication result, and (**i**) The overall fabrication result.

A detailed description of how to extract the vector path from the SVG is diagrammed in Figure 3. Here, a carp structure was used as an example. Many basic components, e.g., ellipse, curves, and circles, can be found in this SVG. When applying the SVG to organize the paths, five steps are required: (1) interpreting the SVG, (2) constructing the outline of the structure, (3) arranging the path for multi-focal two-photon lithography, (4) partition, and (5) generating SLM phase maps.

#### 2.2.1. Interpreting the SVG

An SVG [44,45,46] is a language for describing two-dimensional vector graphics. Vector graphics operate on primitives such as lines, points, curves, and polygons. Unlike the traditional bitmap [47] and Tiff [48], which only contain light intensity distribution information, the SVG also provides path information. It can also be zoomed in or out without distortion or loss of quality.

Each SVG has a corresponding text file to interpret the graph. In the text file, the labels can be interpreted as different mathematical equations to represent the curves using a computer.

**Figure 4:**
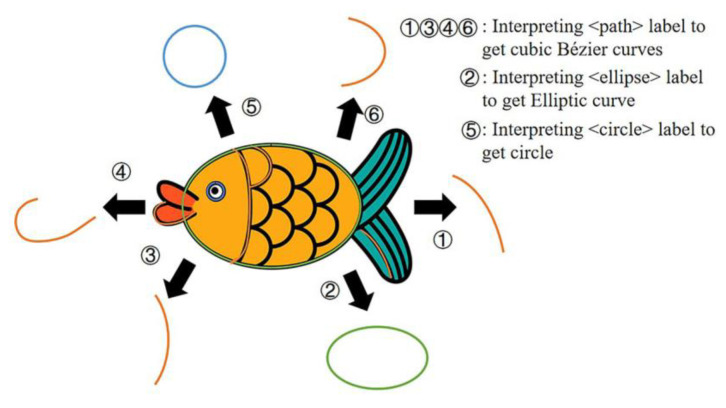
Constitution of a carp SVG as an example.

One typical vector graphic is plotted in Figure 4. Generally, the carp structure comprises a head, a body, scales, and a tail. The elliptic body is expressed by the <ellipse> label, which is represented by an elliptic function as follows:

(4) $$\frac{{\left(x-x_{c}\right)}^{2}}{a^{2}}+\frac{{\left(y-y_{c}\right)}^{2}}{b^{2}}=1$$

where $\left(x_{c},\;y_{c}\right)$ is the coordinate of the center point of the ellipse, and a and b are the long and short axes of the ellipse, respectively.

The head, scales, and tail are expressed by <path> labels with cubic Bézier curves, which are represented by the cubic Bézier curve function $B\left(t\right)=\left(x,y\right)$, where

(5) $$B\left(t\right)=B_{0}{\left(1-t\right)}^{3}+3B_{1}t{\left(1-t\right)}^{2}+3B_{2}t^{2}\left(1-t\right)+B_{3}t^{3},\;t\in \left[0,1\right]$$

where $P_{0}$ is the coordinate of the start point in the cubic Bézier curve; $P_{1}$ and $P_{2}$ are the coordinates of two control points, respectively; $P_{3}$ is the coordinate of the termination point in the cubic Bézier curve; and $P_{i}=\left(x_{i},y_{i}\right)$ with i=0, 1,2,3, which are the 2D coordinates. For instance, the code <path class = “cls-2” d = “M58.49,10.79c7.41,0,13.43,5.3,13.43,11.83” transform = “translate(0 8.81)”/> represents a cubic Bézier curve, with the coordinates $P_{0}=\left(58.49,\;10.79\right)$, $P_{1}=\left(7.41,\;0\right)$, $P_{2}=\left(13.43,\;5.3\right)$, and $P_{3}=\left(13.43,11.83\right)$, respectively.

The labels and the corresponding structures are summarized in Table 1. By interpreting the labels, the outline of the shape to be fabricated can be determined.

#### 2.2.2. Outline of the Structure

Using the carp structure as an example, Figure 3a shows the original SVG carp pattern. Figure 3b shows the outline of the simulated carp after interpreting the labels. The mouth, scales, and tail of the carp were plotted using the cubic Bézier curve. The outlines of the eyes and body of the carp were plotted using circles and ellipses, respectively. It can be seen that the outline of the carp is consistent with the SVG.

#### 2.2.3. Arrangement of the Path for Multi-Focal Two-Photon Lithography

The outline must be transferred to discrete fabrication points for further processing in SLM. This process is crucial for determining the fabrication efficiency and quality, which are determined by a series of parameters, including the size and resolution of the fabrication. If the resolution of the fabrication is high, it is difficult to achieve a large fabrication area by SLM without moving the translation stage, instead requiring a partition fabrication, as elucidated in next subsection.

For high-resolution fabrication, the full width at half maxima (FWHM) of the multi-focal spots must be small, with small intervals (Δd) between the fabrication points as well. According to the Nyquist–Shannon sampling theorem [49], Δd≤d/2, where d=0.61λ/NA is the FWHM of the laser spot, with λ and NA being 800 nm and 1.25, respectively. The larger the Δd, the faster the fabrication; however, the quality may be worse. In this investigation, Δd≤d/2 to compromise fabrication efficiency and quality, with the actual value of Δd being no larger than 0.195 μm.

Relying on the vector feature of the SVG, sequential arrays of $X\left(l\right)$ (horizontal coordinates), $Y\left(l\right)$ (vertical coordinates), and their corresponding relationship arrays $P\left(l\right),$ which indicates the index of the next fabrication point, were established. Here, l represents the lth fabrication point, $1\leq l\leq N_{coor}$. $N_{coor}$ is the total number of the fabrication points. For example, if $P\left(3\right)=8$, the 8th fabrication point $(\left(X\left(8\right),\;Y\left(8\right)\right)$ will be fabricated right after the third fabrication point $(\left(X\left(3\right),Y\left(3\right)\right)$. The $P\left(l\right)$ array is generated according to the SVG.

When a structure from the SVG is fabricated in parallel with multi-focal spots, $N_{spot}$ is defined as the number of Bessel–Gaussian laser spots; then, there are $N_{spot}$ laser direct writing paths, correspondingly. After the structure is discretized to $N_{coor}$ fabrication points, the fabrication points of $\left[1,Ngroup\right]$ are assigned to the first laser spot, where $N_{group}=ceil\left(N_{coor}/N_{spot}\right)$. Here, $ceil\left(\cdot \right)$ denotes a ceiling function. Then, the fabrication points of $\left[N_{group}+1,\;2N_{group}\right]$ are assigned to the second laser spot, and so forth.

#### 2.2.4. Partition

In SLM-based LDW, the multi-focal spots can be moved by SLM only without moving the translation stage. However, the movement range of the multi-focal spots is determined by the optical system. If a high fabrication resolution is required, we need to use a high NA objective lens. Accordingly, the range of multi-focal spots that can be moved by SLM is quite limited. For instance, to fabricate structures with an O (100 nm) resolution, an objective lens of NA=1.25 is applicable. Restricted by the small diameter of the objective lens aperture, the movement range of multi-focal spots is only ±1.5 μm, which is relative to the center of the modulation field. Thus, if a large target area is to be fabricated, the original fabrication points must be divided into partitions before generating SLM phase maps, as shown in Figure 3d.

The number ($N_{part}$), size, and position of the partitions should be determined according to the performance of the optical system and the area of target. During partition, all $N_{coor}$ fabrication points are assigned to each partition (Figure 3e), re-assigned as $X_{i}\left(l\right)$, $Y_{i}\left(l\right)$ and $P_{i}\left(l\right)$, which are as follows:

(6) $$\begin{array}{c} X_{i}\left(l\right)=X\left(l\right)-X_{C,i}\\ Y_{i}\left(l\right)=Y\left(l\right)-Y_{C,i} \end{array}$$

where $1\leq l\leq N_{coor,\;i}$, i denotes the *i*th partition, and $X_{C,i}$ and $Y_{C,i}$ represent the center coordinates of the ith partition, respectively. If $N_{spot}$ multi-focal spots are modulated in the parallel fabrication, the $N_{coor,\;i}$ fabrication points will be assigned to the $N_{spot}$ as average as possible. Equation (3), in this case, then reads as follows:

(7) $$\psi \left(x_{0},y_{0}\right)=\frac{2\pi }{\lambda }\frac{NA}{Rn_{t}}\left(x_{0}X_{i}\left(l\right)+y_{0}Y_{i}\left(l\right)\right)$$

It should be noted that $P_{i}\left(l\right)$ needs to be re-assigned according to $P_{i}\left(1\right),$ which is normally the fabrication point on the boundary.

In the fabrication of the carp structure, which is 18.10 × 24.56 μm, $N_{spot}=3$ Bessel–Gaussian laser spots were modulated simultaneously. With an NA=1.25 objective lens, each partition was 3 μm × 3 μm. Therefore, the carp was divided into 6×8 partitions, as shown in Figure 3d.

#### 2.2.5. Generating SLM Phase Maps

(A) For targets with a smaller size than the movement range of the multi-focal spots, no partition is needed. The phase maps of multi-focal spots shown in Figure 3f can be generated directly by SLM according to Equation (3) by the following:

(8) $$\begin{array}{c} \Delta x=X\left(l\right)\\ \Delta y=Y\left(l\right) \end{array}$$

Overall, a number of $N_{pm}=ceil\left(N_{coor}/N_{spot}\right)$ phase maps are generated, with $N_{spot}$ fabrication points or fewer in each phase map. Here, $ceil\left(\cdot \right)$ denotes a ceiling function. After loading all of the phase maps in sequences into the SLM, the multi-focal spots move according to the coordinates, as diagramed in Figure 3g. Then, with proper control of the exposure time of the individual phase map, the target can be fabricated in parallel by two-photon lithography.

(B) For targets with a larger size than the movement range of the multi-focal spots, $N_{part}$ partitions are needed. The phase maps are generated according to the index of partition first, i.e., the phase maps in the first partition are generated in sequence. Then, the phase maps in the second partition are generated immediately following the last phase map of the first partition. By repeating the progress above, all of the phase maps can be generated. The generation sequence is as follows:

(9) $$M_{1}\left(1\right),\;M_{1}\left(2\right),\;\cdots ,\;M_{1}\left(N_{pm,1}\right),\;M_{2}\left(1\right),\;M_{2}\left(2\right),\;\cdots ,\;M_{2}\left(N_{pm,2}\right),\;\cdots ,\;M_{N_{part}}\left(1\right),\;M_{N_{part}}\left(2\right),\;\cdots ,\;M_{N_{part}}\left(N_{pm,N_{part}}\right)$$

In each partition, phase maps can be generated according to Equation (7) and follow the steps in case (A). By synchronizing the movement of the translation stage, loading of phase maps, and laser exposure time, the structures in each partition can be fabricated in sequence, as shown in Figure 3h. Finally, a carp structure with a large area was fabricated, as shown in v 3i.

## 3. Experimental Setup

**Figure 5:**
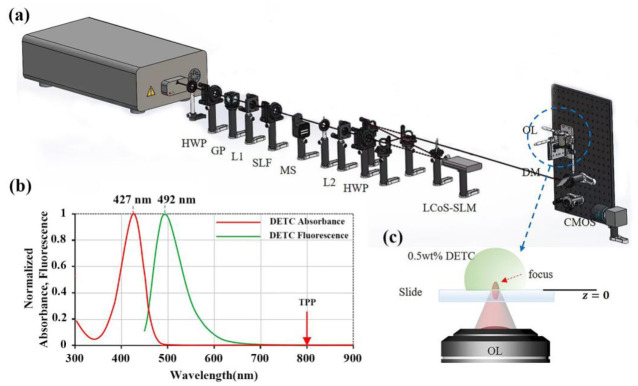
Experimental fabrication with SVG-guided SLM LDW. (**a**) Diagram of the experiment setup. HWP: half-wave plate, GP: Glan prism, SLF: spatial light filter, lenses: L1 and L2 composing a beam expander, MS: mechanical shutter, LCoS: liquid crystal plate on silicon SLM, DM: dichroic mirror, OL: objective lens, R: reflector, and CMOS: CMOS camera. (**b**) Absorbance and fluorescence spectra of 0.5 wt% 7-diethylamino3-thenoylcoumarin (DETC) [50]. (**c**) Schematic of TPP laser writing. z=0 refers to the modulated laser is located at the interface of the photoresist and slide.

The system for SVG-guided SLM LDW is diagramed in Figure 5. The light source was a femtosecond laser (Coherent, Chameleon Ultra II, San Francisco, CA, USA) with a center wavelength of 800 nm. The power of the incident laser was first adjusted by a Glan prism (GP) (Thorlabs, AFS-SF10, Newton, NJ, USA) and a half-wave plate (HWP) (Thorlabs, AHWP05M, Newton, NJ, USA), then filtered by a spatial light filter (SLF) (Thorlabs, P2500UK, Newton, NJ, USA) and collimated by two lenses (L1 and L2) (Thorlabs, LA4130-B, Newton, NJ, USA) to improve the beam quality. A mechanical shutter (MS) (Thorlabs, SH05, Newton, NJ, USA) was applied to accurately control the exposure time of the light source, and the MS response speed was approximately 4.08 ms. The laser subsequently passed through the second HWP (Thorlabs, AHWP05M-980, Newton, NJ, USA), and the polarization direction of the beam was consistent with the long axis of the liquid crystal on silicon (LCoS) SLM (Holoeye, PLUTO-2-VIS-016, Berlin, Germany). The expanded laser was loaded with the corresponding phase information after passing through LCoS-SLM. Then, the modulated laser was reflected through a mirror to a dichroic mirror (DM) (Chroma, ZT532rdc_NIR, Hangzhou, Zhejiang Province, China); after reflecting on the dichroic mirror, the laser was focused into photoresist by a high-NA objective lens (OL) (Olympus, ACHN100XOP 100× NA 1.25, Tokyo, Japan) for LDW. The fabrication process was monitored through a CMOS camera (Daheng Optoelectronics, MER-2000-19U3C-L, Beijing, China) in real time. The movement of the sample stage in the system was controlled by the piezoelectric ceramic nanostage (Pl, P-562.6CD, Karlsruhe, German), which could realize nanometer levels, ideally 2 nm, precise movement with a 200 μm stroke on the *X*-, *Y*-, and *Z*-axes.

In this investigation, the photoresist is a mixture of photoinitiator DETC (Exciton, 1176692, Ohio, USA) and pentaerythritol triacrylate (PETA) (Sigma-Aldrich, San Luis, MI, USA) monomers, which were mixed to a mass ratio of 1:199. After stirring through a magnetic stirrer for 12 h, a clean and transparent yellow-green viscous liquid, i.e., 0.5 wt% DETC, was prepared. As shown in Figure 5b, the photoresist had a wide absorption band around 427 nm and emitted fluorescence around 492 nm. The SVG-guided SLM LDW system adopted inverted microscope configuration. As shown in Figure 5c, the laser passed through the slide from bottom up into the liquid DETC to initiate the polymerization. The axial center of the focal spot was located at the interface between the photoresist and slide in the fabrication, i.e., *z* = 0.

## 4. Results

For the targets with a smaller size than the movement range of the multi-focal spots, no partition was needed, while for the targets with a larger size than the movement range of the multi-focal spots, a partition was needed. Here, we show how the fabrication was carried out and the preliminary results.

### 4.1. Fabrication Results of Concentric Circle and Star Structures in a Small Area

**Figure 6:**
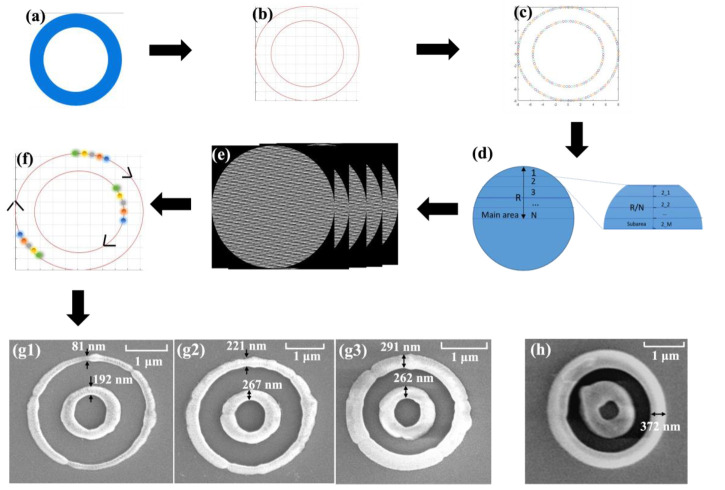
Fabrication of a small–area concentric circle structure with one and three Bessel–Gaussian laser spots, respectively. (**a**) Interpret the SVG into mathematical equations, with a concentric circle pattern as example. (**b**) Construct the outline of the concentric circle. (**c**) Discrete the outline into coordinate points (Δx, Δy). (**d**) The phase maps sequence is generated by the SSP method. (**e**) Generate phase maps with three Bessel–Gaussian laser spots and their movements. (**f**) According to the coordinates through path designing, where the arrows represent the direction of the fabrication path. **(g1–g3**) Final fabrication results with a single Bessel–Gaussian laser spot, where the laser power is (**g1**) 2.6 mW, (**g2**) 3.3 mW, and (**g3**) 4 mW respectively. (**h**) Final fabrication result with three Bessel–Gaussian laser spots, where the laser power is 12 mW. Here, d=0.39 μm and Δd= 0.07 μm. The exposure time for a single phase map is 17 ms.

In the first case, a concentric circle structure was fabricated by a single Bessel–Gaussian laser spot to show the feasibility of the beam control in the SVG-guided SLM modeling. The light field modulation range was 3 μm × 3 μm and z=0. Since the FWHM of the laser spot d was approximately 0.39 μm, the interval between the fabrication points Δd was set as 0.07 μm. The exposure time for a single phase map was 17 ms according to the refreshing frequency of the SLM. The fabrication process is illustrated in Figure 6a–f. The laser powers of 2.6, 3.3, and 4 mW at the pupil were adopted and the corresponding fabrication results are shown as Figure 6(g1–g3), respectively. It is obvious that a set of concentric circle structures were successfully fabricated by the method with a laser power of 2.6 mW. The minimum line width in Figure 6(g1) was 81 nm. The line width increased in width to 221 nm as the power increased to 3.3 mW, as shown in Figure 6(g2). Meanwhile, with the power achieved at 4 mW, the increasing width of the line slowed down and the line width of the structures reached 291 nm.

These results show that the laser positioning was accurate and the scanning path was complete through the whole fabrication. Therefore, SVG-guided SLM modeling has obvious advantages in the fabrication of curved structures.

Subsequently, the concentric circle structure was fabricated simultaneously by three Bessel–Gaussian laser spots. Here, we only wanted to validate the feasibility of the method; thus, more laser spots were not applied. In this case, since the FWHM of the laser spot d was approximately 0.39 μm, the interval between the fabrication points Δd was set as 0.07 μm. The light field modulation range was still 3 μm × 3 μm. The laser power was 12 mW and the exposure time for a single phase map was also 17 ms. The fabrication results are shown in Figure 6h. Although concentric circle structures were fabricated, the width of the structure was apparently larger, relative to that by a single laser spot. The minimum width was 372 nm. In the fabrication of inner circles, the structures were nonuniform with worse fabrication quality, relative to that by a single laser spot. However, the uniformity of the outer circles in Figure 6h is better than that of Figure 6(g1–g3). From Figure 6(g1–g3,h), it can be seen that, above a distance from the fabrication center, uniform and high-resolution fabrication with either single of multi-focal spots can be achieved simultaneously.

**Figure 7:**
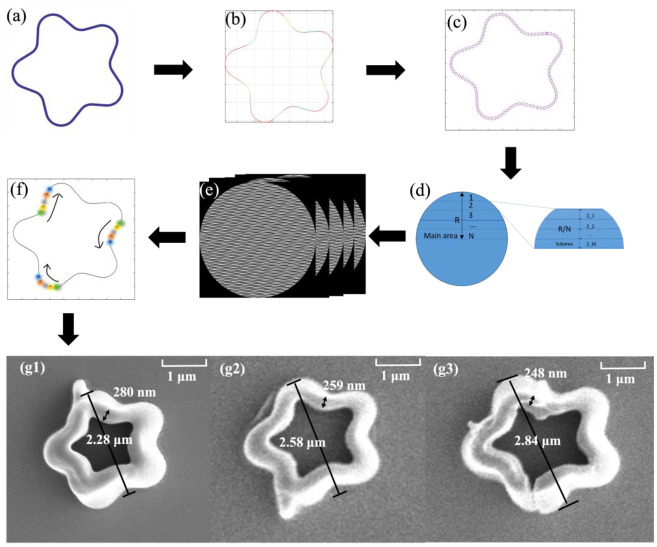
Fabrication of a small–area star structure with three Bessel–Gaussian laser spots. (**a**) Interpret the SVG into mathematical equations, with a star pattern as example. (**b**) Construct the outline of the star. (**c**) Discrete the outline into coordinate points (Δx, Δy). (**d**) The phase maps sequence is generated by the SSP method. (**e**) Generate phase maps with three Bessel–Gaussian laser spots and their movements. (**f**) According to the coordinates through path designing, where the arrows represent the direction of the fabrication path. (**g1–g3**) Final fabrication results with three Bessel–Gaussian laser spots. Here, d=0.39 μm and Δd= 0.07 μm. The laser power is 10 mW and the exposure time for a single phase map is 17 ms. The sizes of the structures in (**g1–g3**) are 2.28, 2.58, and 2.84 μm, respectively.

A relatively more complex structure—a star, which is described by cubic Bézier curve—was also fabricated by the three Bessel–Gaussian laser spots. The FWHM of the laser spot d was approximately 0.39 μm and the light field modulation range was 3 μm × 3 μm. The interval between the fabrication points was Δd= 0.07 μm. The power was 10 mW and the exposure time for a single phase map was 17 ms. Here, three star structures of different sizes were fabricated, as shown in Figure 7(g1–g3). As shown in Figure 7(g1–g3), as the size of the fabricated star structure increased from 2.28 to 2.84 μm, the fabrication resolution increased gradually, with a decreasing width to 248 nm. It can be seen that at the same laser power, if the size of the star structure was larger, the width of the star structure was smaller, i.e., a high fabrication resolution.

### 4.2. Fabrication of a Carp Structure in a Large Area

**Figure 8:**
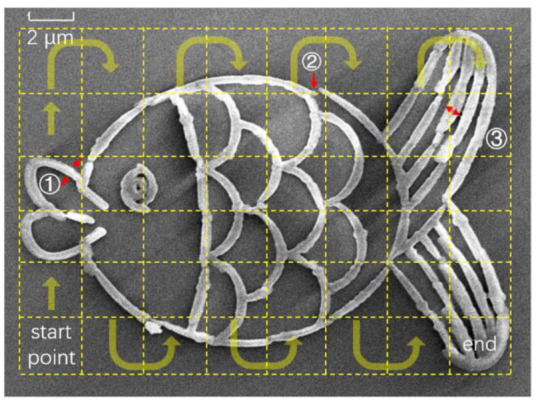
Fabrication of a large–area carp structure with three Bessel–Gaussian laser spots. The size of the carp structure was 18.10 μm × 24.56 μm. Here, d=0.39 μm and Δd= 0.07 μm. The laser power was 13.5 mW and the exposure time for a single phase map was 17 ms. The fabrication process follows the yellow arrow from the start point to the end. The red arrows and its corresponding serial numbers ①, ②, ③ indicate the problem observed in fabrication.

In this section, the fabrication of a carp structure using SVG-guided SLM LDW is introduced. It had an area of 18.10 μm × 24.56 μm, which is larger than the modulation area of 3 μm × 3 μm; therefore, a partition was needed. Here, we still used three Bessel–Gaussian laser spots, with d=0.39 μm and Δd= 0.07 μm. The exposure time for a single phase map was 17 ms. Under a laser power of 13.5 mW, a large-scale carp pattern was implemented with a typical width of 420 nm. The fabrication result is shown in Figure 8, with the carp equally segmented into six rows and eight equally. The sequence of the fabricating areas is shown by the yellow arrows. From mark ①, it can be seen that the SLM modeling guided by SVG could implement the TPP laser system to fabricate a continuous curve structure of high quality at the beginning of the fabrication. In fact, during the fabrication of the whole structure in Figure 8, all of the curved structures had a smooth morphology, which is also one of the characteristics of this method. Meanwhile, at mark ②, there was a break at the joint point, which may have been caused by insufficient exposure at the joint point, resulting in the break and dislocation of the structure during development. Finally, the line width of the whole pattern gradually increased through the fabrication; for example, the line width reached 730 nm at mark ③, which was likely caused by the defocus of the laser spots.

### 4.3. Solid-Structure Fabrication

**Figure 9:**
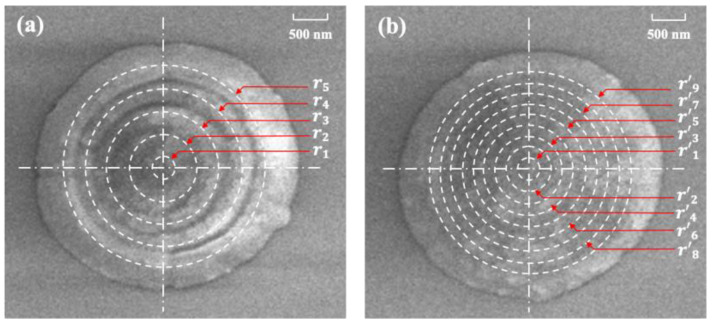
Fabrication of a solid structure by diminishing contours with SVG-guided SLM LDW. Circular structure fabricated by (**a**) 5 and (**b**) 9 diminishing contours.

Generally, the SVG-guided SLM LDW is capable for solid-structure fabrication. For a 2D-filled surface, if the structures have no requirement of the z-directional resolution, this can be approached by adjusting the phase maps to fabricate a series of contours of similar structures of smaller sizes. As shown in Figure 9, a circular-shaped surface can be implemented by fabricating a series of concentric circles with different radii. In Figure 9a, the radii from $r_{1}$ to $r_{5}$ were 0.166, 0.499, 0.832, 1.16, and 1.49 μm, respectively. In Figure 9b, the radii from $r{’}_{1}$ to $r{’}_{9}$ were 0.166, 0.332, 0.499, 0.665, 0.832, 0.998, 1.16, 1.33, and 1.49 μm, respectively. It can be seen that a 2D solid structure could be easily implemented by diminishing the contours. The quantity of the contours depends on the laser power and the size of the target structure. For a 3D structure, the simplest method is fabrication layer by layer. By adjusting the z-position of the target and repeating the 2D fabrication steps, a three-dimensional structure can be obtained.

## 5. Discussion

Laser fabrication based on the SVG-guided SLM should be more efficient and time-saving compared to the progressive scanning in conventional LDW. Assume that the scanning area in LDW consists of M×N points, among which the target structure contains A points. Let $t_{p}$ be the exposure time at each fabrication point, $t_{m}$ be the required time of the translation stage moving from the previous point to the next point, and $t_{s}$ be the response time of the optical switch. Then, the fabrication time of the target structure consists of two parts. One part is the time taken to fabricate the structure with A points, i.e., $t_{1}$, while the other part is the time taken to move the stage over the points of M×N−A, i.e., $t_{2}$. Since $t_{1}=t_{p}+t_{m}+t_{s}\times A$ and $t_{2}=t_{m}\left(M\times N-A\right)$, the time taken to fabricate the target structure with progressive scanning is $T_{1}=t_{1}+t_{2}$.

Meanwhile, in SVG-guided SLM LDW, laser focus is only generated on the path of the target structure to avoid unnecessary scanning of the structureless region. Assume that the target structure consists of *A* fabrication points as well. Let $t_{r}$ be the exposure time of the focal spots, $k_{spot}$ be the number of the focal spots, and *S* be the quantity of the sub-region in the scanning area. Then, the fabrication time of the structure can be written as $T_{2}=A\times t_{r}/k_{spot}+t_{m}+t_{s}\times S$, ($k_{spot}>1$).

Take the fabrication system in this paper as an example; the parameters are as follows:

$$t_{p}=t_{r}\approx 17\;\mathrm{ms},\;t_{m}\approx 5\;\mathrm{ms},\;t_{s}\approx 3\;\mathrm{ms}$$

For the carp pattern in Figure 8, S=48 and $k_{spot}=3$. Then, $T_{1}/T_{2}\approx 33$, that is, when fabricating the same structure, the traditional progressive scanning method is approximately 33-fold longer than the SVG-guided SLM LDW.

On the contrary, SVG-guided SLM LDW has a higher fabrication speed and is more competent for constructing complex structures compared to ordinary SLM-based laser multi-focus fabrication. For example, in Zhu’s work [34], in order to fabricate the pattern of “NWU”, the coordinate information of each point needs to be manually calculated at first. Then, the coordinates are substituted into the calculation program to generate corresponding phase maps, which are loaded into SLM to implement parallel fabrication. However, in SVG-guided SLM, all of the coordinate points of the fabrication path are directly generated by computer interpretation and discretization of SVG. Subsequently, the phase maps for the parallel fabrication are carried out automatically. Obviously, it is more time-saving and efficient than ordinary SLM-based parallel fabrication for all fabrication processes that are planned and scheduled automatically.

A higher refreshing speed in SLM will inevitably increase the fabricating speed. However, due to the limitations of phase map transmission and the response time of liquid crystal molecules, the current refresh speed of mainstream commercial SLM is 17 ms.

**Figure 10:**
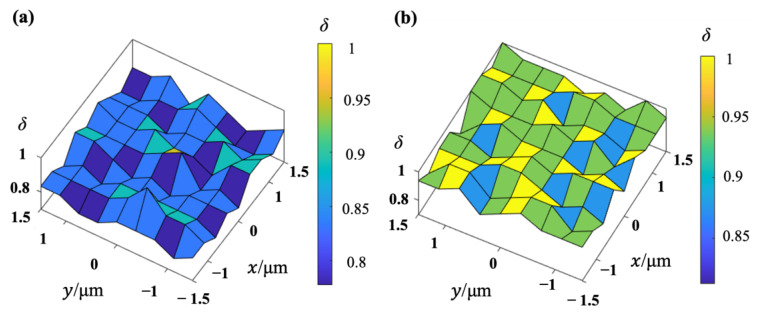
Distribution of the modulation efficiency at different input laser powers: (**a**) 3 mW and (**b**) 4 mW.

Although the SVG-guided SLM LDW approach advanced in this investigation was proven to be effective, from the fabrication results, some problems still exist. For example, it can be seen from Figure 6(g1–g3) that the line width of the inner circle is slightly wider than that of the outer circle, especially with a low laser power. This indicates that the modulation efficiency ($\delta =P_{m}/P_{0}$) of the modulated laser spot at different positions might be different ($P_{0}$ and $P_{m}$ are the laser powers before and after modulation, respectively). Figure 10 shows the modulation efficiency of the laser spot at different positions. It can be seen that the change in the SLM efficiency for the laser focuses within the fabrication range is quite small, which means that the intensity and morphology of the laser focus at different fabrication positions are stable. Therefore, the different line widths between the inner and the outer circles is likely caused by overlapping of the laser spots during the fabrication. Specifically, the interval between the two fabrication points, Δd, refers to the corresponding path length in the fabrication track. Since the inner circle has a larger curvature than the outer circle, the overlap between the fabrication points is larger than that of the outer circle when fabricating the inner circle. Therefore, the line width of the inner circle is wider than that of the outer circle. This phenomenon is more obvious in the case of fabrication with low power, as shown in Figure 6(g1). With an increase in the laser power, the greater the size of the laser fabrication voxel achieved at a certain value; thus, the line width of the inner and outer circles gradually converge, as shown in Figure 6(g3), (h). Similarly, in Figure 7(g1–g3), the larger star structure has a smaller structure width.

## 6. Conclusions

In this investigation, we developed a novel LDW method by combining two-photon absorption, SLM, and a vector path guided by SVG (SVG-guided SLM LDW) for fast, flexible, and parallel nanofabrication. As a preliminary test of the method, three laser focuses were independently controlled with three different paths to optimize fabrication and promote time efficiency. In the fabrication with a single laser spot, a minimum width of 81 nm was reached. Meanwhile, for the fabrication with three laser spots, accompanied by a translation stage, a carp structure of 18.10 μm × 24.56 μm was fabricated with a typical structure width of 420 nm. Although the current results are not perfect, indicating the method is still far from being fully developed, the method shows the potential to change the inherent lithography method, making it possible to efficiently fabricate complex structures. The current method highlights the possibility of developing LDW techniques toward fully electrical systems and realizes the lithography technology of engraving complex structures quickly and efficiently.

## Figures and Tables

**Table 1:** Coding information of an SVG.

Label	Parameter	Function	Graphs and Curves
<rect>	Width		Rectangle
	Height		
<circle>	Circle center coordinates ($x_{c}$, $y_{c}$)	${\left(x-x_{c}\right)}^{2}+{\left(y-y_{c}\right)}^{2}=r^{2}$	Circle
	Radius r		
<ellipse>	Coordinates of the center of the ellipse ($x_{c}$, $y_{c}$)	$\frac{{\left(x-x_{c}\right)}^{2}}{a^{2}}+\frac{{\left(y-y_{c}\right)}^{2}}{b^{2}}=1$	Ellipse
	Horizontal radius a		
	Vertical radius b		
<polygon>	Start coordinates ($x_{1}$, $y_{1}$)	Line 1: $y=\frac{y_{2}-y_{1}}{x_{2}-x_{1}}\cdot \left(x-x_{1}\right)+y_{1}$ Line 2: $y=\frac{y_{3}-y_{2}}{x_{3}-x_{2}}\cdot \left(x-x_{2}\right)+y_{2}$ …… Line n: $y=\frac{y_{n}-y_{1}}{x_{n}-x_{1}}\cdot \left(x-x_{1}\right)+y_{1}$	Polygon
	($x_{2}$, $y_{2}$)….. ($x_{n}$, $y_{n}$) $\left(n\geq 3\right)$		
	End coordinates ($x_{n}$, $y_{n}$)		
<line>	Start coordinates $(x_{1}$, $y_{1})$	$y=\frac{y_{2}-y_{1}}{x_{2}-x_{1}}\cdot \left(x-x_{1}\right)+y_{1}$	Line
	End coordinates $(x_{2}$, $y_{2})$		
<polyline>	Start coordinates $(x_{1}$, $y_{1})$	Line 1: $y=\frac{y_{2}-y_{1}}{x_{2}-x_{1}}\cdot \left(x-x_{1}\right)+y_{1}$ Line 2: $y=\frac{y_{3}-y_{2}}{x_{3}-x_{2}}\cdot \left(x-x_{2}\right)+y_{2}$ …… Line n−1: $y=\frac{y_{n}-y_{n-1}}{x_{n}-x_{n-1}}\cdot \left(x-x_{n-1}\right)+y_{n-1}$	Polyline
	$(x_{2}$, $y_{2})\ldots (x_{n-1}$, $y_{n-1})$$\left(n\geq 3\right)$		
	End coordinates $(x_{n}$, $y_{n})$		
<path>	M/m	$(x_{0}$, $y_{0})$	Initial coordinate of outline
	L/l	Line: $y=\frac{y_{2}-y_{1}}{x_{2}-x_{1}}\cdot \left(x-x_{1}\right)+y_{1}$	Straight line
	V/v	Line: x=a	Vertical line
	H/h	Line: y=a	Horizontal line
	C/c S/s	$x=x_{0}{\left(1-t\right)}^{3}+3x_{1}t{\left(1-t\right)}^{2}+3x_{2}t^{2}\left(1-t\right)+x_{3}t^{3},\;t\in \left[0,1\right]$ $y=y_{0}{\left(1-t\right)}^{3}+3y_{1}t{\left(1-t\right)}^{2}+3y_{2}t^{2}\left(1-t\right)+y_{3}t^{3},\;t\in \left[0,1\right]$	Cubic Bezier curve
	Q/q T/t	$x=x_{0}{\left(1-t\right)}^{2}+2x_{1}t\left(1-t\right)+x_{2}t^{2},t\in \left[0,1\right]$ $y=y_{0}{\left(1-t\right)}^{2}+2y_{1}t\left(1-t\right)+y_{2}t^{2},\;t\in \left[0,1\right]$	Quadratic Bezier curve
	A/a	$x=x_{c}+acos\theta$ $y=y_{c}+bsin\theta$ $\theta \in \left(\theta_{1},\theta_{2}\right)$	Elliptical arc
	Z/z	$(x_{n}$, $y_{n})$	End coordinate of outline

## Data Availability

Not applicable.
